# The Impact of Handheld Device Use on Hand Biomechanics

**DOI:** 10.3390/bioengineering12111145

**Published:** 2025-10-23

**Authors:** Melinda J. Choi, Valeria P. Bustos, Kyle Y. Xu, Vasudev Vivekanand Nayak, Paulo G. Coelho, Kashyap K. Tadisina

**Affiliations:** 1 DeWitt Daughtry Family Department of Surgery, Division of Plastic Surgery, University of Miami Miller School of Medicine, Miami, FL 33136, USA; 2 Department of Biochemistry and Molecular Biology, University of Miami Miller School of Medicine, Miami, FL 33136, USA; 3 Dr. John T. Macdonald Foundation Biomedical Nanotechnology Institute (BioNIUM), University of Miami, Miami, FL 33136, USA; 4 Sylvester Comprehensive Cancer Center, University of Miami Miller School of Medicine, Miami, FL 33136, USA

**Keywords:** carpometacarpal joint, metacarpophalangeal joint, biomechanics

## Abstract

Cell phone use has become ubiquitous in everyday life for many, yet the potential long-term impacts on hand biomechanics remain unknown. A review was performed on the topic of handheld device use and biomechanics of the hand to identify common findings as well as gaps in the literature. A literature search was performed using several databases and a comprehensive search strategy using controlled keywords was designed. A total of 1556 studies were screened, and 28 studies examining handheld device use were included. A total of 2173 individuals participated in the included studies where cell phone (*n* = 23) and tablet (*n* = 5) usage were examined, focusing on the kinematics (*n* = 17), muscles (*n* = 13), joints (*n* = 2), nerves (*n* = 4), and tendons (*n* = 1) of the hand. Handheld device use placed the thumb carpometacarpal (CMC) and metacarpophalangeal (MCP) joints in extreme positions of abduction, as well as wrist extension and ulnar deviation. Increased muscle activity of the first dorsal interossei, extensor digitorum communis, and abductor pollicis brevis was demonstrated while using a handheld cellular device. Studies also suggested that handheld device use is powered by the thumb CMC and MCP joints, as well as intrinsic musculature. Thus, individuals could consider operating handheld devices with a two-hand grip, minimizing device size/weight, or using the index finger or voice texting to decrease muscular fatigue and offload joints. Further studies should be conducted to evaluate the long-term effects of cell phone use on the hand and wrist.

## 1. Introduction

The use of cell phones or handheld devices has become an extension of daily life. As many as 98% of the population in advanced economies possess a mobile phone [1,2]. Within this population, there is a wide proportion of adults aged over 50 who own smartphones (55–96%), in comparison to 98–99% of young-adults who are smartphone owners [2]. This trend in smartphone use is also pervasive in the pediatric population [3,4,5]. Despite how commonplace the use of handheld devices is, a clear understanding of the biomechanical demands on the hand while using a mobile device is not well-defined. Additionally, the relationship between these biomechanical changes and the development of surgical hand pathology has not been described. Moreover, as the number of children and young adults using mobile devices continues to rise, the potential long-term effects on the development of hand pathologies remain uncertain. The aim of this study is to provide a summary of the effect of handheld device use on structures of the hand, specifically joints, muscles, nerves, and tendons, describe the mechanics required when using these devices, and provide a general overview of the associated potential hand pathologies that may arise.

## 2. Methods

### 2.1. Search Methodology

A comprehensive search was conducted using the PubMed, Scopus, Embase, CINAHL, and Web of Science databases. Keywords searched included (biomechanic* OR movement OR proprioception OR kinematic*) AND (text* OR cell phone OR handheld device OR smart phone) with conversion to Mesh terms or database-specific keywords. The use of the word “text” was in the context of “text messaging” or “texting”, as a way of capturing the colloquial terms used for sending and receiving text messages. Because text messaging is one of the more active applications of cell phone use, this terminology was included in the search to capture any relevant studies.

### 2.2. Study Selection

Two reviewers independently performed initial title and abstract screening for inclusion (M.C., V.P.B.). If discordant, a discussion was held with the final decision made by the senior reviewer (K.K.T.). Studies then underwent a full-text analysis. Inclusion criteria included all studies evaluating a structural component of the hand (e.g., bone, tendon, nerve, muscle) while using a handheld device. Exclusion criteria rejected studies that only evaluated clinical diagnoses of the hand, did not evaluate a hand structure while using a handheld device, or abstracts.

### 2.3. Data Extraction and Synthesis

Data extraction was performed on the included studies. Relevant information, including year of publication, population size, study design, measured outcomes, assessment of one or both hands of individuals, and type of handheld device, was extracted. Of note, data involving muscles of the shoulder or upper arm was excluded. To elaborate, while proximal muscle data would affect hand biomechanics, the typical clinical scenario in which patients present with hand or forearm related complaints would not lead to meaningful interventions for the clinician who is charged with managing the distal upper extremity. This study sought to provide clinical insight to the hand and upper extremity surgeon who may more frequently encounter these patients and complaints.

## 3. Results

### 3.1. Study Characteristics

A total of 1556 studies were identified once duplicate studies were removed. A total of 36 studies were then subjected to full-text analysis. After full-text screening, a total of 28 studies met the eligibility criteria and were included for data extraction. In total, the included studies pooled 2173 individuals (Table 1).

Subjects were studied while using a phone (*n* = 23) or a tablet (*n* = 5). Outcomes were assessed either unilaterally (*n* = 22) or bilaterally (*n* = 6). Studies evaluated kinematics (*n* = 16), muscles (*n* = 13), joints (*n* = 2), nerves (*n* = 4), and tendons (*n* = 1) of the hand. Studies that evaluated the kinematics and joints of the hand specifically evaluated the position of the thumb carpometacarpal (CMC), metacarpophalangeal (MCP), and interphalangeal (IP) joints. The wrist joint was also evaluated (*n* = 8). Of the studies that evaluated the muscles of the hand, extrinsic muscles only (*n* = 3) or both intrinsic and extrinsic hand muscles (*n* = 10) were analyzed. Two nerve studies examined the median nerve of individuals while they were dynamically using the hand. One study evaluated the flexor pollicis longus (FPL) and median nerve in a static position. In all studies, participants were observed using either real handheld devices or mock devices with measurable keys. Study designs, including methods and measured outcomes, were variable among the studies.

### 3.2. Kinematic and Joint Studies

Studies evaluated joint position and thumb or wrist kinematics with motion capture systems to measure the position of respective joints. Markers were placed on joints of interest, and a motion capture camera recorded the position of these markers. The relative position of the joints could then be calculated, and data processed according to preferred or established models. Joint angles were either measured directly with a goniometer or through calculations from biomechanical models based on motion capture data.

CMC joints were noted to generate the greatest joint reaction forces while using a handheld device when compared to the IP and MCP joints [8]. With regard to specific motions, thumb CMC abduction and adduction powered the movement from top right to bottom left of cell phones, as well as top to bottom movement [14,15]. CMC flexion and extension, however, powered the movement in the opposite direction, from top left to bottom right. Additionally, with the thumb reaching towards the bottom right of the phone, the wrist, IP, and MCP joints were flexed, and the thumb CMC was flexed and pronated [14,15,24]. In contrast, with the thumb reaching towards the top left of the phone, the wrist was extended and more adducted, and the thumb CMC was extended and supinated [14,15]. CMC abduction was greatest while reaching for the bottom left of the phone. While texting, the thumb was held in abduction at the CMC joint, and the MCP and IP joints were flexed [25].

Different-sized devices were assessed, with findings that the wrist was less extended when holding smaller tablets [20] or while holding a tablet in portrait orientation versus landscape orientation [24]. Further, the wrist was noted to be more ulnarly deviated while holding the tablet in landscape orientation [24]. While using tablets, the thumb CMC had the greatest amount of abduction while reaching for the bottom left of the device [24]. With swiping motions on the tablet, horizontal swiping employed greater MCP and CMC joint abduction compared to vertical swiping [24].

The grip on devices was also evaluated. A two-hand grip on a cell phone produced greater wrist extension, thumb CMC extension, abduction, supination, and significantly more thumb MCP extension than a one-hand grip [17,28]. A one hand grip on a tablet led to wrist radial deviation compared to a two-hand grip [31]. However, while typing on a tablet, wrists were significantly more ulnarly deviated [31].

### 3.3. Muscle Studies

All studies evaluated muscles with the use of electromyograms (EMGs). Muscles assessed among all studies included first dorsal interossei (FDI), abductor pollicis longus (APL), extensor pollicis brevis (EPB), abductor pollicis brevis (APB), extensor digitorum communis (EDC), flexor carpi radialis (FCR), flexor carpi ulnaris (FCU), flexor digitorum superficialis (FDS), flexor pollicis brevis (FPB), extensor carpi radialis (ECR), and extensor carpi ulnaris (ECU). EMG activity was recorded while performing specific tasks that either directly involved using a handheld device or simulated the hand motions employed during handheld device use. APB and FDI activity were increased when the thumb was in planar abduction [7]. APB activity increased when the thumb moved from an adducted to an abducted posture, while FDI activity increased when the thumb moved from an extended to a flexed posture [9,16]. ECR activity was higher when texting with one thumb compared to a two-thumb texting technique [32].

The hand holding smaller tablets showed less EDC activity relative to larger tablets [20,24]. EDC activity was significantly lower when the tablet was held in portrait orientation compared to landscape orientation in the dominant holding hand [20]. However, when evaluating the ECR and ECU activity of the swiping hand, activity was greater with portrait orientation [24]. Overall, however, ECU and EDC activity were greater than FCR and FCU activity while typing on a tablet [31].

Pertaining to hand size, individuals with small hands demonstrated greater muscle fatigue with device use [9]. Smaller hands also showed greater FDS and EDC activity [21]. Females have been reported to demonstrate greater EDC and APL activity when texting compared to males [29]. Individuals who tended to use a handheld device with high thumb velocity demonstrated greater EDC and APB muscle activity when compared to individuals with lower thumb velocity [18,19]. Further, FDI activity was higher for narrow phones compared to wider phones [21].

### 3.4. Nerve and Tendon Studies

Two nerve studies evaluated the median nerve with ultrasound visualization while individuals performed cell phone-related tasks. In a study that simulated cell phone use, the cross-sectional area of the median nerve decreased over time and activity with real-time measurements [13]. However, a study that measured the median nerve immediately after rapid typing on a cell phone demonstrated a significant increase in cross-sectional area without a change in the flattening ratio [22]. Both studies showed the median nerve dynamically moved in a longitudinal plane with device use [13,22].

One study specifically evaluated the flexor pollicis longus (FPL) tendon. In static ultrasound evaluations, the FPL tendon cross-sectional area of high-frequency users was significantly higher at the mid-thenar and MCP joint [26]. Additionally, the median nerve was found to have a larger cross-sectional area in the dominant hand compared to the non-dominant hand in these high-frequency smartphone users [26,33].

## 4. Discussion

Handheld device use has altered the biomechanics of hand performance. The thumb has been traditionally considered the stabilizer of the hand while grasping or performing activities. However, the use of handheld devices has flipped this paradigm and forced the thumb to take on a more dexterous role while other digits have stabilized grip [8]. For example, the index finger has taken on the role of the primary grip finger when a phone is held [16,21]. Further, a two-hand grip has been shown to improve stability by separating the dual task of holding and operating the device [17,19]. Thus, much of the discomfort that arises from the use of handheld devices can be related to the need to stabilize the device with inherently non-stabilizing digits, while also requiring users to perform multiple functions with one hand.

### 4.1. Joints and Kinematic Findings

Given the critical role of the thumb in operating handheld devices, joint studies have demonstrated that much of thumb control is driven by the more proximal joints. When the thumb CMC joint performs abduction/adduction movements, the IP and MCP joints are considered static [14,15]. Thumb flexion and use at the extreme reaching position of the cell phone was driven by the CMC and MCP joints [17,24]. Using the thumb with a tablet also demonstrated that most joint excursion was from the thumb CMC joint [30]. One study demonstrated no significant difference in performance in cell phone use with the thumb IP fixed at 10 or 30 degrees [10], suggesting that the IP joint was not a critical determinant in functional cell phone use.

In a study evaluating performance while moving the thumb in different directions, movement from the top right to bottom left of cell phones resulted in the best performance [14,15]. Given CMC abduction/adduction powers this motion, this suggested the stability and demand of this specific thumb movement [14,15]. In contrast, with the thumb moving toward the bottom right of the phone, the balance of the phone was maintained with wrist flexion and finger flexion, which was a more complex motion and placed thumb joints at their limits of range of motion, while also worsening performance [14,15,25]. Additionally, larger phones constrained thumb CMC joint movement, resulting in a diminished range of motion and lower precision [6,14,15]. Ultimately, thumb CMC joint motion tends to power most of the thumb during device use, and CMC abduction/adduction is a more stable and effective pattern of motion. Clinicians should be suspicious for potential thumb CMC pathology in patients presenting with thumb pain, especially associated with prolonged handheld device use. Further, voice texting or texting using the index finger could be considered to decrease load on the thumb.

Regarding the wrist, position was variable while using a handheld device. Observational studies have noted the wrist was typically not in a truly neutral position [12,31]. Wrist flexion was determined to be relatively close to neutral during cell phone use, and the degree of flexion was lower than the angles associated with high carpal tunnel pressure [27]. From our clinical experience, while wrist pathology may not develop as quickly as thumb pathology due to the smaller relative load, prolonged amounts of wrist deviation is hypothesized to lead to earlier signs of ECR or ECU tendinitis in patients, and needs to be explored in forthcoming studies.

### 4.2. Muscle Findings

In evaluating the muscles of the hand, the level of muscular exertion required during handheld device use was low relative to the maximum voluntary contraction of each muscle, ranging from <5% to ~20% [21,27,31]. This suggests that the use of handheld devices in itself does not require a significant amount of muscular strength [23]. With prolonged activity, however, fatigue can develop.

Studies demonstrated the critical role of intrinsic musculature to operate handheld devices, and the role of the FDI was thought to stabilize the grip of a device [7,18]. When the thumb performed precise motions, such as typing, the greater accuracy required recruited effort from the FDI for stabilization, thus increasing its activity when the thumb moved from an extended to a flexed posture [7]. Individuals felt more uncomfortable moving the thumb in extension and flexion compared to moving the thumb in adduction and abduction. This may be related to the greater control required of the thumb or increased exertion of FDI. Translated to hand surgical implications, the importance of recognition and diagnosis of compressive neuropathy of the ulnar and median nerves cannot be understated, as the intrinsic muscles are first impacted with prolonged nerve ischemia caused by compression.

Additionally, extrinsic extensors also played an important role during handheld device use. Individuals with high thumb velocity demonstrated higher EDC activity, thought to be related to the need for greater wrist stabilization when using the thumb quickly [18]. One study identified females having greater EDC and APL activity compared to males, likely due to the greater thumb abduction and wrist stabilization required for relatively smaller hand sizes [29]. The use of a one-hand grip to operate a handheld device compared to a two-hand grip demonstrated higher ECR and EDC activity, possibly related to the increased stabilization needed to support the device in one hand [19,27,32]. Further, with increased device weight, FDS, ECR, and EDC activity was increased, due to the increased moment arm created by a heavier device, requiring greater muscular activity to support the device weight [20,24,32]. This could contribute to fatigue with a greater recruitment of muscular energy to operate heavier devices.

These findings demonstrate the high recruitment of intrinsic muscles FDI and APB to move the thumb, as well as the role of EDC and ECR in stabilizing the wrist while simultaneously holding the device. Extended usage may lead to symptoms associated with excessive thumb abduction, necessitating additional assessment. Patients may present with signs of tendinitis or De Quervain’s tenosynovitis and could be counseled on mitigating the frequency of aggravating motions. These findings may also encourage mobile device users to consider voice texting or index finger use to decrease fatigue of the intrinsic and radial-sided extrinsic muscles.

### 4.3. Nerve Findings

In evaluating the median nerve, static evaluations of the median nerve demonstrated the enlargement of the cross-sectional area, while dynamic studies show mixed outcomes. When observing the median nerve while typing on a cell phone, the cross-sectional area of the nerve increased after the task was performed [22]. In simulated cell phone use, the cross-sectional area of the median nerve decreased over time during activity [13]. It is possible that the median nerve was being compressed during activity with rebound swelling once the activity was stopped. Regardless, both dynamic studies demonstrated the longitudinal motion of the median nerve during activity, which is thought to occur in order to prevent severe compression of the nerve [13]. While the additive effects of computer and device use on the median nerve have not been studied, these may play a role in the development of carpal tunnel symptoms or other nerve compressions, warranting robust evaluation in clinical settings.

### 4.4. Limitations

This review is limited to conclusions based on the method of data extraction. The included studies varied in study design (such as the duration of exposure), studied populations, and measured outcomes. Therefore, it was not feasible to draw conclusions of significance from these studies. Patterns, common findings, and confounding factors (such as age, sex, hand size, and pre-existing conditions) that could influence the relationship between device use and biomechanical changes are therefore limited in significance. Additionally, many of these studies did not evaluate individuals over a period of time. Therefore, conclusions regarding the long-term biomechanical changes or adjustments in users could not be made. While the included studies did not explicitly evaluate a pediatric age group, likely due to the inability to consent for these types of studies, only five studies evaluated individuals aged 18–19 years old. Given the significant lack of biomechanical studies available involving the pediatric population, this area of future research should certainly be addressed, as handheld device use continues to expand to the younger population.

### 4.5. Clinical Insights and Future Directions

The impact of handheld device usage on hand biomechanics remains a topic requiring thorough investigation. With the widespread usage of these devices and studies suggesting the critical role of the thumb CMC and MCP joints, as well as the increased load placed on intrinsic hand muscles and extrinsic extensors, the development of chronic conditions may manifest earlier for individuals with heavy handheld device usage. An increase in thumb CMC arthritis, carpal tunnel syndrome, and tendinitis may also arise and warrant surgical intervention following more rigorous scientific evaluation.

To reduce mechanical stress, placing the device on a stable surface rather than continuously supporting it with one hand could be explored. This simple ergonomic adjustment is hypothesized to decrease the load on the intrinsic muscles of the hand and wrist. Moreover, considering the complex biomechanics involved in gripping and operating handheld devices (including thumb abduction and adduction, finger flexion and extension, and wrist pronation and supination) it may be beneficial to implement preventative or rehabilitative strengthening protocols. Specifically, targeting the intrinsic hand muscles (e.g., lumbricals, interossei), thenar muscles, and wrist stabilizers may help improve endurance and reduce the risk of repetitive strain injuries—a topic for future exploration.

However, it is important to recognize that these recommendations need to be tailored to the individual’s clinical presentation. Not all patients would benefit from the same approach, and decisions should always be made in the context of a full biomechanical and functional assessment. Just as in many aspects of medicine, these insights are intended to provide clinical context rather than prescriptive rules, emphasizing the need for individualized care plans.

## 5. Conclusions

Overall characterization of the biomechanics of the hand while using handheld devices has been described from varying points of view. These include joint and kinematic studies, EMG studies, and nerve studies. The aim of this paper was to review these studies and identify common themes that emerged. Thumb motion could be attributed to the thumb CMC and MCP joints, with lower contribution from the thumb IP joint. Within the scope of the studies included in this review, intrinsic musculature and extrinsic extensors were major factors responsible for device stabilization and demonstrated the greatest activity during device use. Individuals could therefore consider operating handheld devices with a two-hand grip and/or minimizing the size and weight of the device to decrease muscular fatigue. Alternatives to thumb texting such as voice texting or index finger use may also be considered.

## Figures and Tables

**Table 1:** Study Characteristics.

Study #	Authors, Year [Reference]	Sample Size	Mean or Median Age ^a^	Sex	Laterality Studied	Type of Handheld Device ^b^	Time Exposed with Handheld Device	Metric
1	Xiong et al., 2016 [6]	48 ^c^	youth: 23.6 y/ elderly: 67.5 y	youth: 12 M, 12 F/ elderly: 12 M, 12 F	U	Smartphone: iPhone 4 and Galaxy S4	NS	Kinematic
2	Chang et al., 2017 [7]	50 ^d^	pI: 24.2 y/ pII: 23.6 y	pI: 10 M/ pII: 21 M, 19 F	B	Touchscreen device: MiMo UM-720S	pI: 3.9 y/ pII: 4.2 y	Muscle, Kinematic
3	Kim et al., 2019 [8]	26 ^e^	e: 24.4 y/ Co: 25.1 y	e: 9 M, 10 F/ Co: 7 M	U	Smartphone: Model NS	NS	Joint
4	Chany et al., 2007 [9]	10	25 y	5 M, 5 F	U	Small cellular clamshell phone: LG VX440 Traditional office phone: Panasonic KX-T3165	NS	Muscle
5	Yao et al., 2012 [10]	25	29.8 y	14 M, 11 F	U	Smartphone: Palm Treo	NS	Kinematic
6	Gustafsson et al., 2012 [11]	56	19–25 y	N/A	U	Electrogoniometer: Biometrics SG 110 Surface electromyograph: MuscleTester ME 3000P8	NS	Muscle, Kinematic
7	Gold et al., 2012 [12]	859 ^f^	18–early 20 y	335 M, 524 F	U	Cellphones, Smartphones: Model NS	NS	Kinematic
8	Woo et al., 2016 [13]	30	21.3 y	15 M, 15 F	U	Simulated cell phone usage	NS	Nerve
9	Trudeau et al., 2012 [14]	10	27 y	5 M, 5 F	U	Smartphone: iPhone 3	NS	Kinematic
10	Trudeau et al., 2012 [15]	20	25 y	15 M, 5 F	U	Small, flip, large cell phone and PDA designs ^g^	NS	Kinematic
11	Xiong et al., 2014 [16]	20	24.5 y	10 M, 10 F	U	Smartphone: iPhone 4	NS	Muscle
12	Trudeau et al., 2016 [17]	10	27 y	5 M, 5 F	U	Smartphone: iPhone 3	NS	Kinematic
13	Jonsson et al., 2011 [18]	15	22 y	8 M, 7 F	U	Cell phone: Nokia 3310	NS	Muscle
14	Gustafsson et al., 2011 [19]	60 ^h^	19–25 y	17 M, 24 F ^i^	B	Cell phone: Nokia 3310	NS	Muscle
15	Pereira et al., 2013 [20]	30	30 y	15 M, 15 F	U	Touchscreen device: Non-functional tablet models similar to iPad 2, Kindle Fire, and Samsung Galaxy Note.	NS	Muscle, Kinematic
16	Lee et al., 2016 [21]	120 ^j^	f: 22.6 y/l: 22.3 y	f: 53 M, 37 F/l: 11 M, 19 F	B	Smartphone: Personal (NS) and Experimental (Pantech, Inc. Vega LTE-A)	At least 3 y	Muscle
17	Lai et al., 2014 [22]	31	21.7 y	M 20, F 11	U	Cell phone: Sony Ericsson K750i	NS	Nerve
18	Gustafsson et al., 2018 [23]	19	30.1 y	7 M, 12 F	U	Cell phone: Nokia E5 Smartphone: iPhone 3 GS	NS	Kinematic
19	Coppola et al., 2018 [24]	16	24.5 y	8 M, 8 F	B	Smartphone: Samsung Galaxy III	NS	Muscle, Kinematic
20	Eapen et al., 2018 [25]	234 ^k^	18–29 y	Ca: 58 M, 59 F/ Co: NS	U	Cell phone: Model NS	Ca: 31 Mo/ Co: 28.9 Mo	Joint
21	İnal et al., 2015 [26]	102 ^l^	Non-users: 20.5 y, Low users: 22 y, High users: 20 y	Non-users: 14 M, 22 F/ Low users: 11 M, 23 F/ High users: 5 M, 27 F	U	Cell phone: Model NS	Low users: 24 Mo, High users: 24 Mo	Nerve, Tendon
22	Ko et al., 2016 [27]	27	28 y	M 15, F 12	U	Smartphone: Sony Xperia P	NS	Muscle, Kinematic
23	Sampath et al., 2022 [28]	137	20 y	66 M, 71 F	U	Smartphone: Model NS	3 y	Kinematic
24	Gustafsson et al., 2010 [29]	56 ^m^	19–25 y	Co: 8 M, 7 F/ Ca: 17 M, 24 F	U	Cell phone: Nokia 3310 Personal cell/smart phone: Model NS	NS	Muscle
25	Asakawa et al., 2017 [30]	18	19–42 y	9 M, 9 F	U	Touchscreen device: Samsung Galaxy Tab 2	NS	Kinematic
26	Young et al., 2013 [31]	15	29 y	7 M, 8 F	U	Touchscreen device: iPad 2 and Motorola Xoom	NS	Muscle, Kinematic
27	Kietrys et al., 2015 [32]	20	21.2 y	4 M, 16 F	B	Cell phone: LG 900G Smartphone: iPod Touch, Touchscreen device: Samsung Galaxy Tab GT-P1010 and iPad 2	NS	Muscle, Kinematic
28	Labeeb et al., 2021 [33]	109 ^n^	23–35 y	e: 37 M, 37 F/ Co: 16 M, 19 F	B	Smartphone: Model NS	e: 61 Mo Co: 16.6 Mo	Nerve

Abbreviations: B, bilateral; F, female; M, male; Mo, months; NS, not specified; U, unilateral; y, years. ^a^ Age range provided if mean or median age is unavailable. ^b^ Cell phone: mobile device with QWERTY or alphanumeric keypad inputs, and smartphone: mobile device with touchscreen input, unless otherwise specified. ^c^ Two groups: youth and elderly. ^d^ This study tested two different research questions through two phases (Phase I, pI; Phase II, pII). ^e^ Two groups: smartphone users, exposure group (e) and non-smartphone users, control group (Co). ^f^ Three observers used the Mobile Device Postural Assessment Tool on 859 college-age individuals actively typing on their mobile devices. ^g^ Small: Siemens S56 candy bar; flip: Samsung SCH-i600; large: iMate smartphone; PDA (personal digital assistant): HP iPAQ h4155 pocket computer. ^h^ Two groups: 45 subjects with musculoskeletal symptoms in neck and 15 without symptoms. ^i^ Out of the subjects with musculoskeletal symptoms, three were excluded due to inflammatory disease, and one more excluded due to the use of the index finger rather than thumb to type. Number of male and female participants reported only for the subjects with musculoskeletal symptoms. ^j^ Two groups: field studies (f) and laboratory studies (l). ^k^ Two groups: thumb pain, cases (Ca) and no thumb pain, controls (Co). Of the 117 participants in the Co group, 10 refused ultrasound evaluation. ^l^ Participants were grouped depending on their level of smartphone use: smartphone non-users, high smartphone users, and low smartphone users based on the Turkish version of the Smartphone Addiction Scale. ^m^ Two groups: healthy group, controls (Co) and participants with symptoms, cases (Ca). ^n^ Two groups: smartphone users, exposure group (e) and non-smartphone users, control group (Co).

## Data Availability

No new data were created or analyzed in this study.
